# Army Correspondence—Rambles in Military Hospitals

**Published:** 1863-02

**Authors:** R. M. Lackey

**Affiliations:** Surgeon 98th Reg. Ill. Vol.; Camp near Murfreesboro, Tenn.


### ARMY CORRESPONDENCE.

### RAMBLES IN MILITARY HOSPITALS.

By R. M. LACKEY, M.D., Surgeon 98th Reg. Ill. Vol.

Nashville and its institutions have undergone vast changes since the State of Tennessee was plunged into rebellion. The halls of learning are turned into hospital wards, and where the student and young scholar toiled up the hill of Science, sick and wounded soldiers now find a resting place during the period of their affliction. No department of learning now *flourishes* in this once gay, peaceful little city, but the medical department of the University of Nashville, does *barely* maintain an existence; its number of students has dwindled from three to four hundred, or a baker's dozen. The large college building, a fine structure, is used as a military hospital; the Prof. of Surgery, a man well known, and distinguished for his professional attainments, like the large number of students, is not there; like the glory of the city and her institution, he has departed.

Prof. Bowling is still there, and lectures to the baker's dozen, once or twice a week. Several other medical gentlemen of the city lecture to this small class, making in all six lectures during the week, instead of six a day, as is customary.

There are twenty-three military hospitals in Nashville. Some of the buildings occupied as hospitals are tolerably well adapted—better, I think, than those in any other place that I have visited, and the hospitals are generally well conducted; wards are well ventilated, everything clean and well arranged for comfort and convenience.

Large numbers of those wounded at the battle of Stone's River, have been removed to Nashville. There are still large numbers of those most severely wounded, in Murfreesboro, and in hospitals near the battle field. Deaths from secondary hemorrhage, brought on while being transported in ambulances, are quite common. Two cases of this kind occurred in the ward in charge of my friend, Dr. Hanson, Ass't Surg. 42d Reg. Ill. One was a case of flesh wound, by musket ball, of the thigh, severing a branch of the profunda femoris artery. There had been but little nemorrhage from the time the wound was first dressed, until he was placed in an ambulaece, to be taken to Nashville; while being transported thirty miles over rough roads, a large quantity of blood was lost. The other case was a wound of a branch of the right carotid. The ball entered at the left angle of the mouth, and came out a little below the mastoid process of the temporal bone, on the opposite side. There was but little hemorrhage, and no unfavorable symptoms occurred while he remained quiet. While on the road to Nashville, hemorrhage occurred as in the other case. Ligature of the external iliac and common carotid arteries was performed on these cases, respectively, after their arrival at the hospital, but the loss of blood before the operation, had been so great that the men never rallied.

The mortality after secondary capital operations is, as is usual, very great. I have not met with a single case of secondary amputation of the thigh that has been successful. In two cases of gunshot wound, involving the knee-joint, where an operation was performed, one died about the 21st day, the other case the man was still living when I last heard from him, but there was little or no hope of his recovery, either with or without the operation.

Tetanus is of common occurrence among those wounded in the late battle. In some instances, it attacks those who have received slight flesh wounds of some portion of the body. I have heard of no cases occurring, since the battle, that have recovered. It is not surprising that the disease is of frequent occurrence, when we reflect that the men are suffering from the two most frightful causes of the disease, viz: Cold and wet, and bodily injuries.

Erysipelas is very troublesome, especially in Murfreesboro, where large numbers of wounded are crowded together.

The use of Bromine as a remedy in Erysipelas, seems, in the estimation of some surgeons, to rob the disease of its terrors, and it is to be hoped that the high expectations of it as a remedy in this disease, will not be disappointed. It has been used in the military hospitals in Louisville, Ky., and it is said, by many intelligent surgeons, with uniform success. Prof. Goldsmith, I am informed, was the man who first introduced it into the hospitals there as a remedy in this disease. The beneficial effects of the remedy are obtained by fumigating the atmosphere of the wards where the disease occurs. It is used also locally,—applied so that the fumes come in contact with the diseased surface. Some Surgeons have used it, by inhalation, as a remedy in diphtheria, and with very satisfactory results. I have not seen the remedy used in a sufficient number of cases with benefit, to speak so confidently of its efficacy as some do.

The present location of this army is very unfavorable for health. The country for some miles around Murfreesboro is rather level so that some of the camps must necessarily be on flat places; the heavy rains have made ponds and brooks all around, and in the midst of some of the camps. The old tents are not water proof by any means, so that we sometimes get wet from the clouds above and the earth beneath.

Camp Fever prevails to a considerable extent. The new regiments suffer most from it. Pneumonia, with typhoid condition, ranks next in fatality, according to my observation. Diarrhœa prevails to a greater extent here, than it did at this season last year, in the army in Missouri. At no other time during the past eighteen months, have I met with so many cases of "obstinate nostalgia" among soldiers, some of them, when they can not get to their earth-homes, contract disease, by depression of spirits and filthy habits, and eventually go to their long homes.

Camp near Murfreesboro, Tenn., Feb. 2, 1863.

---

☞ Business letters to the Journal should be addressed to Dr. E. INGALS, Chicago, Ills., P. O. Drawer 5787. When money is received a receipt will be returned with the next number of the Journal, and subscribers failing to get such receipt will confer a favor by giving us notice of the omission.

---

Vaccine Matter.—Fresh and reliable Vaccine Matter may be obtained by enclosing One Dollar and addressing

Chicago Medical Journal,
P. O. Drawer 5787, Chicago, Ill.

